# Exploring the phytoremediation potential of plant species in soils impacted by gold mining in Northern Colombia

**DOI:** 10.1007/s11356-024-35853-8

**Published:** 2025-01-21

**Authors:** Leonomir Córdoba-Tovar, Siday Marrugo-Madrid, Libia Pérez Castro, Eunice Ester Tapia-Contreras, José Marrugo-Negrete, Sergi Díez

**Affiliations:** 1 Environmental Toxicology and Natural Resources Group, Universidad Tecnológica del Chocó, A.A. 292 Quibdó, Chocó Colombia; 2 Universidad de Córdoba, Cra 6 # 76 – 103, Montería, 230002 Córdoba, Colombia; 3 Universidad de Sucre, Cra 28 # 5-267, 700001 Sincelejo, Colombia; 4 Environmental Chemistry Department, Institute of Environmental Assessment and Water Research, IDAEA-CSIC, 08034 Barcelona, Spain

**Keywords:** Metal(loid), Mercury, Plants, Bioconcentration, Translocation

## Abstract

Contamination of soils with toxic metals poses significant threats to human health and ecosystems. Plant-based remediation strategies can play a vital role in mitigating these risks, and the use of plants as a remediation strategy can help reduce these risks. In this study, we investigate the remediation potential of native plants in accumulating and translocating metal(loid)s at a Colombian site impacted by gold mining. The remediation capacity is evaluated using the translocation factor (TF) from roots to shoots and the bioconcentration factor (BCF) from soil to roots. Metal(loid) concentrations in the soil followed the order: Fe > As > Hg > Cd > Pb > Zn > Mn > Cu. In plant tissues, Hg showed higher accumulation in leaves (3.5 mg/kg) compared to roots (2.8 mg/kg). Pb (17.7 mg/kg), As (3.8 mg/kg), Fe (2.5 mg/kg) and Cd (1.2 mg/kg) concentracions were also higher in roots. Metal concentrations in the stems, were generally below 1.0 mg/kg, except for Pb (15.0 mg/kg) and Hg (1.0 mg/kg). The highest BCF values for Hg were observed in *Spondias mombin* L. (18.7), *Cecropia peltata* L. (8.3) and *Gliricidia sepium* (Jacq.) Walp (4.4). On the other hand, *Senna alata* (L.) Roxb., *Psidium guajava* L. and *Morinda citrifolia* L. exhibited notable BFC values for As with 44.7, 6.3 and 5.9, respectively. *Musa x paradisiaca* L. had the highest BCF for Cd (1.8). *M. citrifolia* (4.3) and *Annona muricata* L. (3.2) exhibited the highest TF for Hg, while *Tabebuia rosea* (Bertol.) Bertero ex A.DC. (4.9) and *Paspalum fasciculatum* Willd. ex (3.1) demonstrated elevated TF values for Pb. In conclusion, plants such as *P. fasciculatum*, *A. muricata*, *M. citrifolia*, *G. sepium* and *T. rosea* exhibit great potential for application in phytoremediation strategies in tropical regions impacted by gold mining activities.

**Supplementary Information:**

The online version contains supplementary material available at 10.1007/s11356-024-35853-8.

## Introduction

Human activities like agriculture, timber harvesting, livestock, and mining drive a country's development but also raise global environmental concerns (Biswas and Biswas 2018; Salvarrey et al. 2014). Mining, in particular, is under scrutiny for causing significant environmental changes, including elevated toxic metal concentrations in soil, posing threats to biodiversity and human health (Marrugo-Madrid et al. 2021; Biswas and Biswas 2018; FAO/WHO 2018).

Mining activities are known to elevate metal concentrations, including pollutants like mercury (Hg), lead (Pb), and arsenic (As), often exceeding permissible levels (Sahoo et al. 2018; WHO 1996). Additionally, Importantly, these elements lack any essential role in organism development, representing non-essential metals (Lata and Asthana 2018). Other elements including copper (Cu) and zinc (Zn) play an important role in plant growth in particular, however, high concentrations could impose toxic effects on a given organism (Lewis et al. 2001). Manganese (Mn) is another element that functions as a micronutrient in plants, but elevated concentrations could become a critical environmental problem (J. Liu et al. 2014).

Another problem caused by metals is the impact of the biological processes they lead to on the microbial communities present in the soil (Sheoran et al. 2010; Singh and Kalamdhad 2011). For example, Cd has the ability to affect proteases, ureases and basic phosphatases (Lorenz et al. 2006). Pb affects catalases, acid phosphatases and invertase (Belyaeva et al. 2005). Other enzymes such as sulfatase and phosphatase are affected by As (Lu et al. 2019; Speir et al. 1999), and b-glucosidase can restrict by toxic action of Cu can restrict b-glucosidase (Dussault et al. 2008).

In the Caribbean region of Colombia, there is a documented concern regarding environmental impacts due to gold mining activities (Fuentes-Gandara et al. 2021). In the Mojana floodplain, sediment samples show average mercury (Hg) concentrations ranging from 2.3 to 5.4 mg/kg (Marrugo-Negrete et al. 2015). Furthermore, the presence of 15% bioavailable Hg represents a potential risk to both ecosystem environments and human health. Conversely, in the Boque River (Bolivar Department), sediment concentrations of Cd (1.11 mg/L) and cyanide (1.57 mg/L) exceed permissible limits, imposing a significant threat to both public and environmental health. Additionally, the mutagenic index estimated in this research revealed a high probability of mutations in the surrounding population that consumes this type of water (Martín et al. 2020). Recent soil reports reveal elevated levels of Hg (1.95 mg/kg), Pb (178.7 mg/kg) and Cd (12.7 mg/kg) (Durante-Yánez et al. 2022), and therefore, the need for urgent measures to remediate the soil has been emphasized.

Since some years ago, phytoremediation has garnered significant attention as one of the foremost environmental challenges, regardless of the fact that in some cases remediation technologies remain a challenging and costly process (Araujo et al. 2020). However, phytoremediation is a promising alternative for the remediation of metal-contaminated soils, and it is economically and sustainably viable (Durante-Yánez et al. 2022; Pandey et al. 2015). Phytoremediation is a process that utilizes plants in contaminated soils to mitigate or control the effects of specific metals (Ali et al. 2013). The remediation capacity of a plant has been estimated through the bioconcentration factor (BCF) and translocation factor (TF). In soils highly contaminated with Hg, BCF values have been recorded for species such as *Piper marginathum* L. (0.91), *Cyperus ferax* L. (0.87), *Capsicum annuum* L. (0.83) and *C. peltata* (0.69), making them potential candidates for the restoration of soils contaminated with this metal (Marrugo-Negrete et al. 2016). Concentrations of metals such as Cd, Pb and Zn found in the roots of *Crassocephalum crepidioides* (Benth.) (79.8 Cd µg/kg, Pb 411.3 µg/kg, Zn 1122.8 µg/kg), *Solanum nigrum* Linn. (6.9 Cd µg/kg, Pb 39. 2 µg/kg, Zn 124.8 µg/kg) suggest that these plants exceed the normal ranges and phytotoxic level for these metals and would therefore be effective for soil remediation. Additionally, they show a strong ability to accumulate and transport Cd especially (Zhu et al. 2018).

The use of plants for soil recovery will always be a viable alternative in economic and environmental term remains an economically and environmentally viable alternative (Pandey et al. 2015). Despite advancements in the field, there is still a lack of knowledge regarding the appropriate plants for implementing soil restoration programs at various scales. The phytoremediation capacity of native plants can vary based on factors such as climate, soil, and contamination levels in each region (Hosseinniaee et al. 2022; Khalid et al. 2017). Therefore, the success of phytoremediation programs relies heavily on understanding the specific characteristics of the affected landscape and the phytoremediation potential of plant species (Paes et al. 2023). In this sense, expanding the list of plants and understanding the mechanisms by which plants tolerate a particular metal is fundamental to strengthen and reorient policies aimed at restoring soils contaminated with toxic metals (Sharma et al. 2023).

In this study, we hypothesize that the abundant plant species at a post-mining site could be used to develop phytoremediation activities. Therefore, the objective of this work was to identify plants established in a mining legacy, measure metal(loid) concentrations, and evaluate phytoremediation potential. To achieve this goal, concentrations of metal(loid) (e.g. Hg, As, Cu, Zn, Pb, Cd, Fe and Mn) were determined in both soil samples and various tissues across frequent plant species. Bioconcentration and translocation factors were calculated to aid in the identification of appropriate plants for soil restoration.

## Materials and methods

### General description of the site

**Fig. 1:**
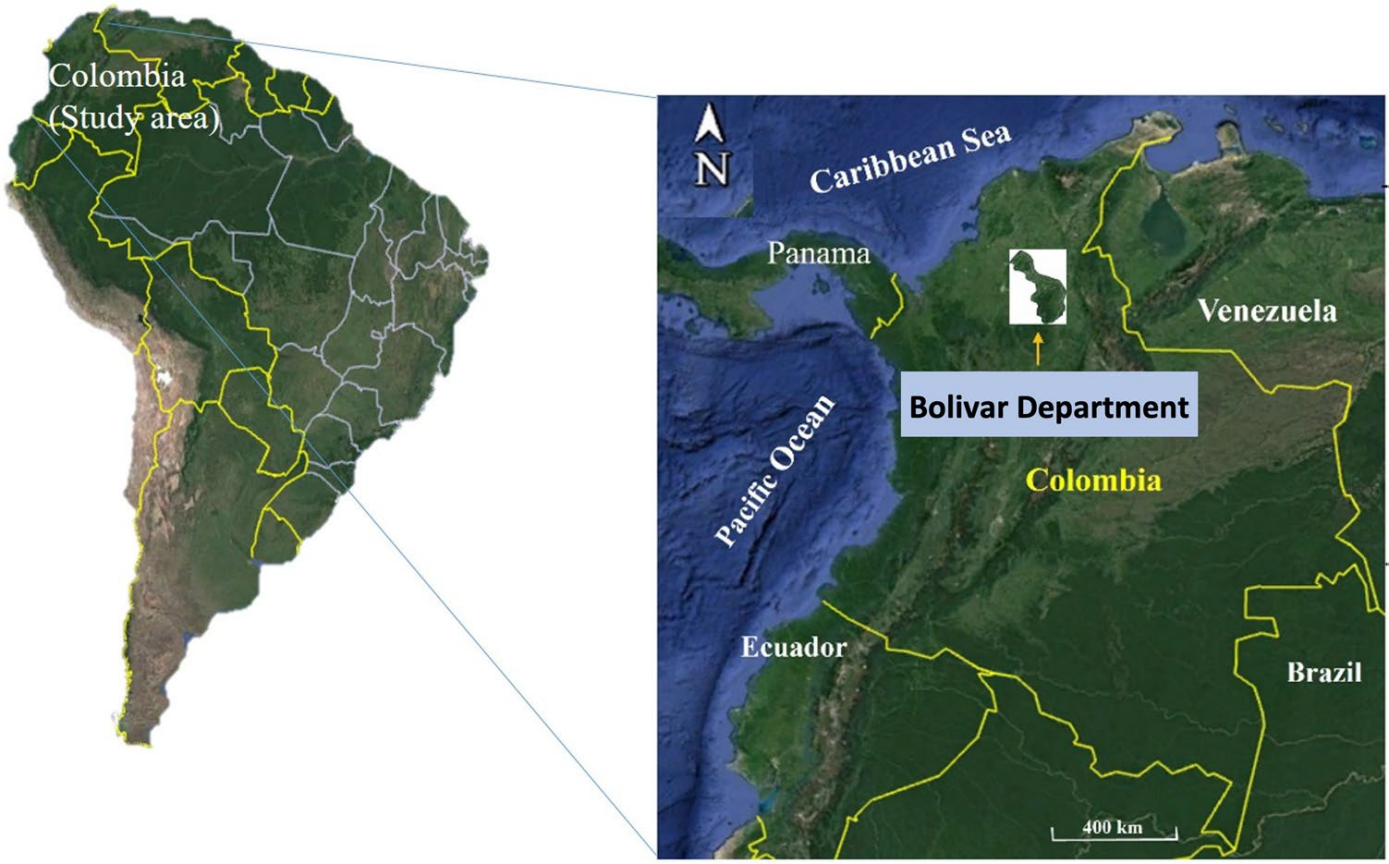
Location of the study area in northern Colombia

The 85% of the region includes undulating relief and low elevation hills associated with lowland coasts. Soils in the region are mostly influenced by continental, Jurassic, Neogene, volcanic, alluvial and marine sedimentary rocks, giving rise to saline, alluvial and sandy soils (Gómez et al. 2020). The study was carried out in the Colombian Caribbean region, focusing on the village of Mina Santa Cruz located in the municipality of Barranco Loba. This is situated in the Department of Bolívar at the following coordinates 08° 56′ 44" N, 074° 6′ 24" W (Fig. 1). Bolívar is characterized as one of the warmest regions in Colombia, experiencing average daily temperatures of 32 °C, relative humidity ranging from 40–60%, and a daily rainfall of 2 mm/day (WorldData 2022).

The municipally of Barranco Loba has a population of 15,186 inhabitants with a density of 36,5 inhabitants/km^2^ distributed over 41,600 ha and is located at 25 m above sea level. The territory is crossed by the Tarazá mountain range in the Cordillera Central and has a dry tropical climate. The dry tropical landscapes in this part of Colombia have outstanding geomorphological features that include mountain ranges, hills, rocky outcrops and piedmont. However, these ecosystems have been subjected to strong anthropogenic pressures including pollution, deforestation, and biodiversity decline (Garzón et al. 2015).

Mining-induced soil contamination is one of the main environmental challenges in the region. In this context, soils near the study site have reported total Hg (T-Hg) concentrations ranging between 0.23 mg/kg and 6.3 mg/kg (Marrugo-Negrete et al. 2016). In addition, soil pH values (3.7- 4.5) and organic matter percentage of 3.2% have been reported at the study site, indicating acid soils with average organic matter contents (Vidal et al. 2010). 

### Sample collection

Between May and September 2018, seven field campaigns were conducted in the study area, during which plant species were collected. For vegetation sampling, twenty 5 × 10 m transects were randomly established covering a total area of 2,000 m^2^ (Kondratenko et al. 2022). Data such as abundance and growth habit (i.e. shrubs, lianas, trees and palms) were recorded visually in each sampling unit. Plant samples were collected, with three specimens for each species (*n* = 3), focusing particularly on the most abundant plants. Most plants were identified in the field, and for those posing identification challenges, taxonomic determination was accomplished through comparison with specimens from the Herbarium of the Universidad Nacional in Bogotá (Colombia). Additionally, to ensure taxonomic changes, we consulted databases, including The Plant List (http://www.theplantlist.org/) and the virtual herbarium of the National University of Colombia (http://www.biovirtual.unal.edu.co/nombrescomunes/es/). Three subsamples were also collected from the topsoil (0–20 cm) under the canopy of each plant until a composite sample was obtained. The samples (plant and soil) were stored in polyethylene bags and transported to the laboratory. Samples were crushed and sieved to < 2 mm to remove stones and roots. They were then stored in sealed, labeled plastic bags in a refrigerator (Marrugo-Negrete et al. 2016; Sun et al. 2016).

### Species composition

The total number of plants collected was represented by 154 individuals grouped in 33 species and 18 botanical families. The vegetation corresponded to a landscape with long periods of natural succession induced by mining activities. The families with the greatest contribution of species were Anacardiaceae > Leguminosae > Melastomataceae > Cyperaceae. Among the abundant species were *T. rosea*, *P. guajava*, *A. muricata*, *Inga edulis* Mart., *P. fasciculatum*, *G. sepium*. In terms of life forms 49% (75 individuals) were shrubs, 48% trees (74 individuals) and finally palms with 3% (five individuals).

### Plant and soil analysis

Plant samples were treated by high-temperature desiccation at 105 ℃ for 30 min. They were then dried in an oven at 65 °C until a constant weight was obtained. The dried plant tissues were ground to a fine powder. All handling procedures were carried out without direct contact with any metal to avoid possible cross-contamination of the samples. The soil pH (1:5 soil—water, w/w) was measured with a conventional pH meter. For the analyses of T-Hg in plants, approximately 0.5 g plant material (dry weight basis, dw) was subjected to assisted digestion by a microwave with a mixture of HNO_3_/H_2_O_2_ (5:2) (Jedrzejczak 2002). Also 0.5 g dw of soil were digested by microwave using 10 mL of 65% HNO_3_ (USEPA 2007). The microwave oven used was a Milestone ETHOS TOUCH 127697 series with a temperature range of 100–175 °C and a pressure of 1500 kPa. The T-Hg was determined by cold vapour atomic absorption spectroscopy (CVAAS) using a Thermo Scientific iCE 3000 series analyser. Analytical quality control of the methods was evaluated in triplicate with certified materials for tomato leaves (CRM 1753a, 34 ng/g), and soil/sediment (CRM008–050, 720 ng Hg/g), and the percentage recovery was 98% and 97%, respectively. The detection limit for T-Hg was 14 ng/g dw, calculated as three times the standard deviation (SD) of the blank (Marrugo-Negrete et al. 2016).

### Bioconcentration factor (BCF) and translocation factor (TF)

In our study, the concentrations found in flowers and fruits were summed over the total aerial part. The factors indicated above were calculated using common formulas that are widely employed in this type of research (Hosseinniaee et al. 2022; Singh et al. 2010; Sun et al. 2016; Yu et al. 2021). The BCF is determined by dividing the metal concentration in aerial parts, primarily in leaves and stems, by the metal content in the soil. On the other hand, the TF is calculated by dividing the metal concentration in aerial tissues by the corresponding metal content in the roots (R. Singh et al. 2010).

### Statistical analysis

In the initial data analysis, normality was assessed using the Kolmogorov–Smirnov test for series over 30 and the Shapiro–Wilk test for data under 30. Descriptive statistics, including mean and standard deviation, were calculated for all concentrations. Values below the detection limit were adjusted to half the limit. Metal concentration differences among plant organs were examined using the Kruskal–Wallis test, with Dunn's post hoc test for multiple comparisons. Additionally, Pearson correlations were conducted, analyzing the relationship between metal concentrations in soil (variable 1) and roots (variable 2) with log-transformed data (Eid and Shaltout 2016).

A principal component analysis (PCA) was used to determine plant behaviour as a function of BCF and TF factors for each metal. The data were standardized to obtain zero mean and standard deviation equal to one. The PCA was accompanied by a cluster analysis (CA) to understand the behaviour of metals in soil. Euclidean distance and Ward's hierarchical clustering measures with two or more groups were used for this analysis. The different metals (Hg, As, Pb, Cu, Zn, Fe and Mn) were coded as variables. PCA and CA were run using Statgraphics Centurion Software (version 16.1). All other analyses were performed in GraphPad Prisma Software (version 8.1) and for all analyses a significance level was defined taking *p* < 0.05.

## Results

### Characteristics of plant species and levels of metal(loid)s in soil and plants

Table 1 describes the composition and characteristics of the plants investigated. The accumulated contents for metal(loid)s in soil and plant tissues of the 33 plants investigated are summarized in Table 2. It should be noted that, unlike the rest of the metals analysed, all plants had a high Hg accumulation response. In *A. muricata* the highest median for Hg was observed in leaves (4.8 mg/kg) as well as in *G. sepium* (2.4 mg/kg) and *I. edulis* (4.4 mg/kg). In *P. fasciculatum* for example, concentrations of Pb (10.1 mg/kg), As (9.6 mg/kg), Cd (0.6 mg/kg) and Hg (0.3 mg/kg) were higher in roots. Cd in *T. rosea* was 0.8 mg/kg in roots and 0.9 mg/kg in leaves. The same species showed concentrations for As of 2.9 mg/kg in roots and 0.9 mg/kg in leaves. However, Pb concentrations were higher in leaves (21.3 mg/kg) compared to roots (6.9 mg/kg).

**Table 1:** List and characteristics of plant species growing in the study area, a dry tropical landscape contaminated by metals in northern Colombia

Family	Species	Life form	n	% species
Anacardiaceae	*Anacardium occidentale* L	Tree	1	0.6%
	*Mangifera indica* L	Tree	5	3.2%
	*Schinus terebinthifolius* Raddi	Shrub	5	3.2%
	*Spondias mombin* L	Tree	5	3.2%
Annonaceae	*Annona muricata* L	Tree	5	3.2%
Arecaceae	*Cocos nucifera* L	Palm	5	3.2%
Bignonaceae	*Crescentia cujete* L	Tree	5	3.2%
	*Tabebuia rosea* (Bertol.) Bertero ex A.DC	Tree	8	5.2%
Bixaceae	*Bixa orellana* L	Tree	1	0.6%
Combretaceae	*Terminalia catappa* L	Tree	5	3.2%
Cyperaceae	*Cyperus longus* L	Shrub	1	0.6%
	*Cyperus sculentus* L	Shrub	5	3.2%
	*Schoenoplectus californicus* (C.A.Mey.)	Shrub	5	3.2%
Leguminosae	*Gliricidia sepium* (Jacq.) Walp	Tree	5	3.2%
	*Inga edulis* Mart	Tree	5	3.2%
	*Senna alata* (L.) Roxb	Shrub	6	3.9%
Malvaceae	*Malachra alceifolia* Jacq	Shrub	1	0.6%
	*Sida rhombifolia* L	Shrub	5	3.2%
Melastomataceae	*Miconia jucunda* (DC.) Triana	Shrub	5	3.2%
	*Miconia lacera* (Bonpl.) Naudin	Shrub	5	3.2%
	*Miconia sp.*	Shrub	5	3.2%
Musaceae	*Musa x paradisiaca* L	Shrub	6	3.9%
Myrtaceae	*Psidium guajava* L	Tree	7	4.5%
Poaceae	*Paspalum fasciculatum* Willd. ex Flüggé	Shrub	5	3.2%
Pteridaceae	*Adiantum sp.*	Shrub	5	3.2%
	*Pteris longifolia* L	Shrub	5	3.2%
Rubiaceae	*Morinda citrifolia* L	Tree	6	3.9%
Rutaceae	*Citrus aurantiifolia* (Christm.) Swingle	Tree	5	3.2%
	*Citrus x limonia* Osbeck	Tree	5	3.2%
Solanaceae	*Capsicum annuum* L	Shrub	5	3.2%
	*Physalis angulata* L	Shrub	1	0.6%
	*Solanum sp.*	Shrub	5	3.2%
Urticaceae	*Cecropia peltata* L	Tree	6	3.9%
Total	33		154	100%

**Table 2:** Descriptive statistics of cumulative metal content (median ± standard deviation, mg/kg) in soil and plant organs of established plants in a dry tropical landscape in northern Colombia

		Plant organ		
Metals	Soil	Root	Stem	Leaf
Hg	18.4 ± 7.2	**2.8 ± 4.5**	1.0 ± 2.6	**3.5 ± 7.4**
Cd	1.7 ± 3.1	1.2 ± 2.6	0.7 ± 1.5	0.2 ± 1.9
As	28.8 ± 11.7	**3.8 ± 4.8**	0.9 ± 0.6	1.5 ± 1.3
Pb	0.2 ± 0.6	17.7 ± 58.5	15.0 ± 30.2	12.9 ± 102.9
Cu	0.03 ± 0.03	0.01 ± 0.09	0.01 ± 0.06	0.09 ± 0.07
Zn	0.1 ± 0.0	0.1 ± 0.09	0.1 ± 0.1	0.06 ± 0.06
Fe	34.5 ± 8.9	**2.5 ± 2.8**	0.2 ± 0.2	0.3 ± 1.7
Mn	0.8 ± 1.2	0.1 ± 0.1	0.2 ± 0.2	0.1 ± 0.2

Values in bold indicate Statistical difference (*p* < 0.05, post hoc test Dunn´s)

Globally, the organs with the highest Hg accumulation were leaves (3.5 mg/kg) followed by roots (2.8 mg/kg). Concentrations for Pb (17.7 mg/kg), As (3.8 mg/kg) and Cd (1.2 mg/kg) were also higher in roots, while the remaining trace metals exhibited lower concentrations in comparison. Flowers and fruits generally exhibited concentrations below the detection limit, although sometimes the concentrations of Hg, Cd, As, Pb and Fe were above the detection level. The variations in metal accumulation indicate that metal accumulation process is influenced by both the plant species and the type of metal.

Pb contents in plants exceeded the permissible limit of 0.03 µg/kg in all plant organs. Cd and As contents in plants were also higher than the permissible values of 0.02 µg/kg and 0.03 µg/kg, respectively (FAO/WHO 1989, 1995).

**Fig. 2:**
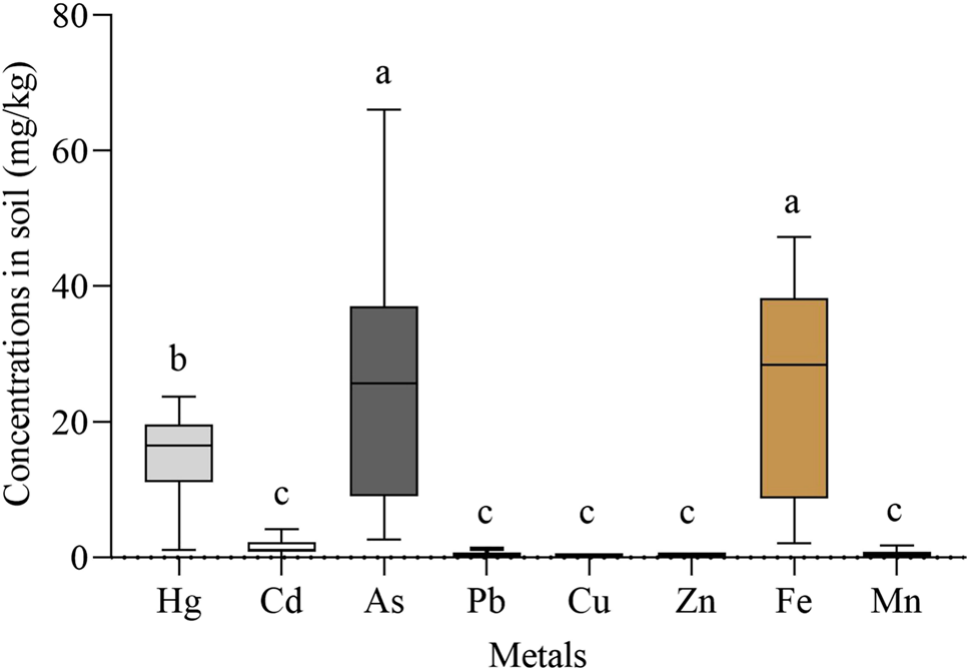
Comparison of medians of metals evaluated in soil using Kruskal–Wallis (post -hot Dunn´s). Medians that do not share a letter are statistically different at a significance level of *p* < 0.05

According to the Kruskal–Wallis test the concentrations of Hg, As and Fe in roots and leaves, when all plants were considered were significant (*p* < 0.05). On the other hand, the concentrations of the remaining metals showed no significant difference between plant tissues (*p* > 0.05). When global contents for all metals in soil were compared, the levels of Hg (16.5 ± 6.5 mg/kg) were statistically significant and different from the rest of metals (Fig. 2). Finally, the contents of all metals in soil except As and Pb were positively correlated with those found in plant roots (Table S1).

### Bioconcentration (BCF) and translocation (TF) factors of metal(loid)s in plants

**Fig. 3:**
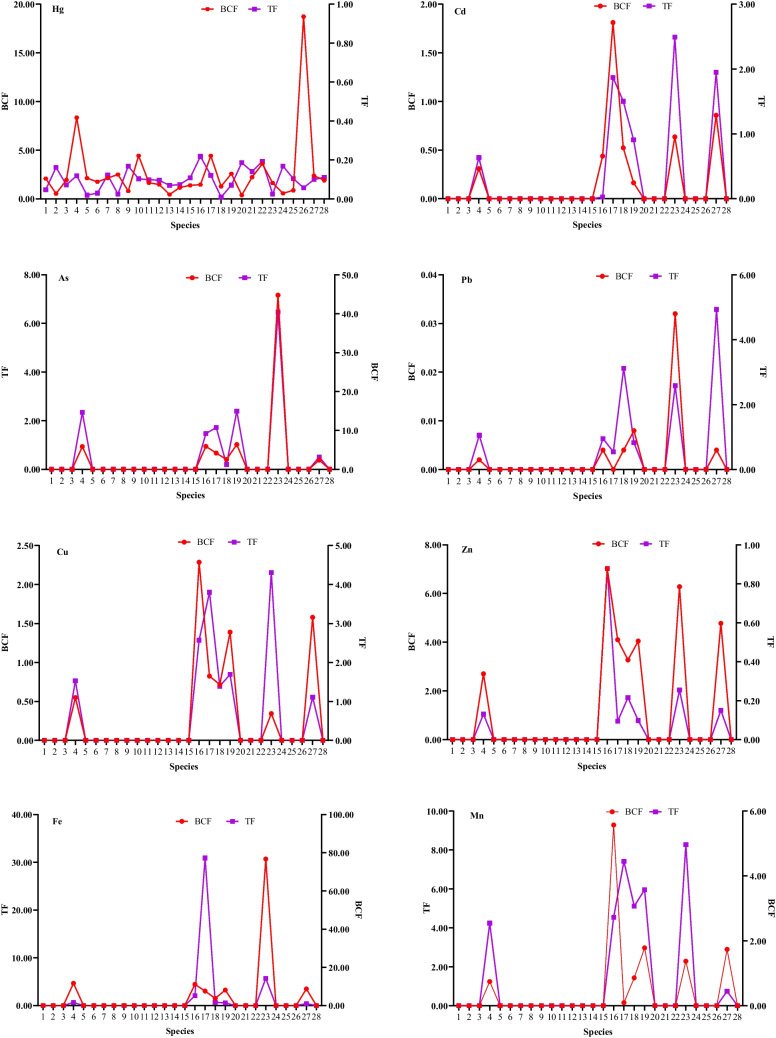
BCFs and TFs of metals for plants collected in the study area (The horizontal ordinate numbers represent different kind of plants as follow: 1. *Adiantum sp*.; 2. *A. muricata*; 3. *C. annuum*; 4. *C. peltata*; 5. *C. aurantiifolia*; 6. *C. limonia;* 7. *C. nucifera*; 8. *C. cujete*; 9. *C. sculentus*; 10. *G. sepium*; 11. *I. edulis*; 12. *M. indica*; 13. *M. jucunda*; 14. *M. lacera*; 15. Miconia sp.; 16. *M. citrifolia*; 17. *M. paradisiaca*; 18. *P. fasciculatum*; 19. *P. guajava*; 20. *P. longifolia*; 21. *S. terebinthifolius*; 22. *S. californicus*; 23. *S. alata*; 24. *S. rhombifolia*; 25. Solanum sp.; 26. *S. mombin*; 27. *T. rosea*; 28. *T. catappa*)

The average value for BCF considering all plants (n ≥ 5) for the case of Hg was 2.6 and 1.9 for TF, Cd (BCF 0.6, TF 1. 3), As (BCF 10.2, TF 2.1), Pb (BCF 0.02, TF 2.0), Cu (BCF 1.1, TF 2.3), Zn (BCF 0.5, TF 2.0), Fe (BCF 18.2, TF 5.8) and Mn (BCF 2.9, TF 3.1). The accumulative potential of the plants evaluated from the BCF and TF factors are summarized in Fig. 3. *S. mombin* (18.7), *C. peltata* (8.3) and *G. sepium* (4.4) had the highest values for BCF in the case of Hg. The highest TF values for Hg were observed in their order in *M. citrifolia* > *A. muricata* > and *Pteris longifolia* L. In the case of Cd, *M. paradisiaca* was the plant with the highest BCF (1.8), followed by *T. rosea* with 0.8. *S. alata* (2.4), *T. rosea* (1.9) and *P. fasciculatum* (1.5) obtained the highest TF values for this metal. *Senna alata* L., *P. guajava* and *M. citrifolia* exhibited the highest BFC values for As with 44.7, 6.3 and 5.9 respectively. *T. rosea* (4.9) and *P. fasciculatum* (3.1) obtained the highest TF values for Pb.

### Principal component analysis for BCFs and TFs

**Fig. 4:**
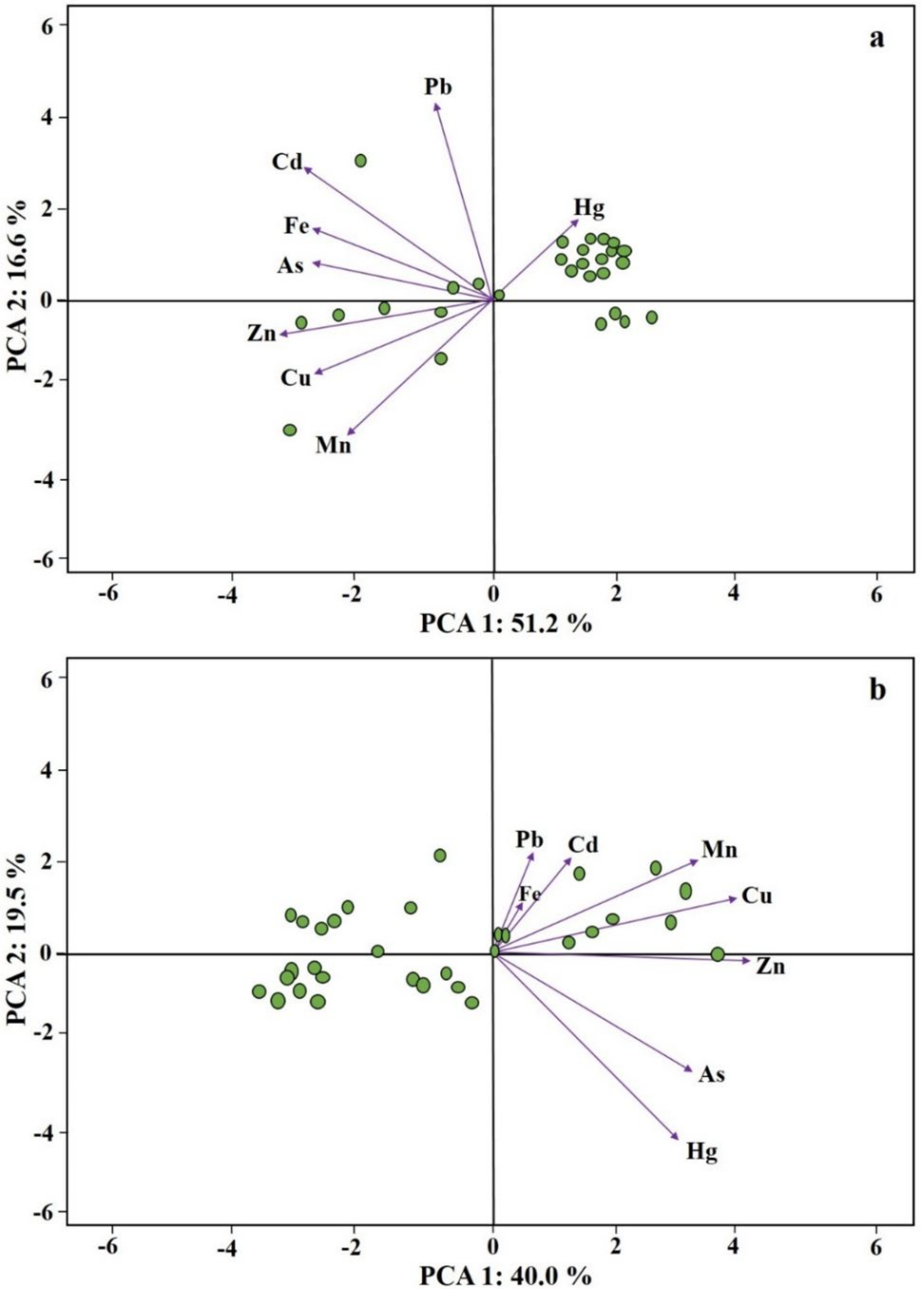
Principal component analysis (PCA) for the BCF (**a**) and TF (**b**) values obtained from the investigated plants

**Fig. 5:**
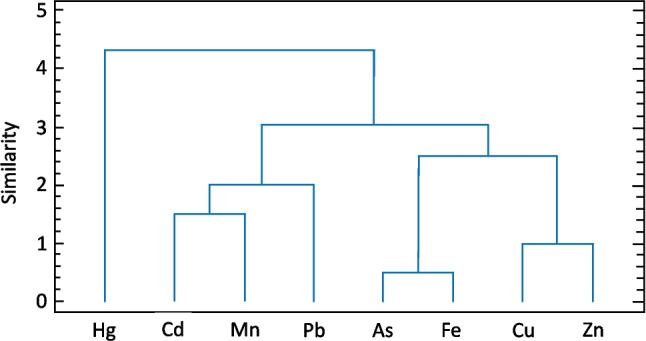
Dendrogram of the cluster analysis showing the results of the hierarchical grouping of the behavior of the eight metals analyzed in soil

The relationship between plants and the BCF and TF factors for each metal was evaluated by principal component analysis (PCA). BCF revealed a positive slope between most plants and Hg contents (Fig. 4a), explaining 67.8% of the variance. In the multivariate analysis, Component 1 demonstrated the highest loading, primarily associated with Hg, explaining 51.2% of the variance. Meanwhile, Component 2 exhibited loadings for Pb, Cd, and Fe, elucidating 16.6% of the overall variability in metal(loid) concentrations. According to the PCA for TF (Fig. 4b), the first two components were able to explain 59.5% of the variance. In the first component, the highest loadings were for Pb and Cd and explained 40.0% of the variance, while PCA 2 explained 19.5% Additionally, cluster analysis revealed a close association between groups of metals (Cu and Zn), (As and Fe) and similar behaviour among them, while Pb, Mn and Cd formed another group (Fig. 5).

## Discussion

Our findings align with other studies, indicating that floristic diversity in fragmented areas is often linked to the tolerance capacity of plant species emerging post-disturbance (de Lima et al. 2021; Domingos et al. 2015). Documenting floristic diversity at a localized scale serves as a valuable tool, aiding scientists in understanding and filling gaps related to anthropogenic impacts on species abundance and richness. This approach contributes to a more comprehensive assessment of environmental changes in fragmented areas (Gillespie et al. 2000).

In this study, shrubs accounted for almost half (49%) of the total number of individuals. These results were similar to those found in the El Alacrán mine in the department of Córdoba where the representation of shrubs had a notorious participation in contrast to trees (Marrugo-Negrete et al. 2016). Similarities were observed in the composition of focal plant species, including *C. peltata*, *S. alata*, *T. rosea* and *C. annum*. Vegetation occurring after disturbance could hold relevant information for the management of remediation of metal-contaminated soils (Gajić et al. 2018; Owusu et al. 2022).

The soil showed contamination by different metals. The highest contents recorded in soil were for Fe (34.5 mg/kg), As (28.8 mg/kg) and Hg (18.4 mg/kg). The concentrations of the metals in soil were positively correlated with the concentrations found in plant roots except for As and Pb. Other studies have also indicated that elements such as Fe, Zn, Mn and Cu have shown a positive correlation between soil concentrations and plant roots (Eid et al. 2019; Eid and Shaltout 2016). In addition, Cd concentrations (1.7 mg/kg) in soil were above the permissible limit value of 0.08 µg/kg (WHO 1996). Because Cd is a highly toxic element, its presence in high concentrations can generate negative effects on human health and on the biocenosis (i.e. biological communities, flora and fauna) present in the ecosystem. This is largely due to the fact that the bioavailability, bioaccessibility and accumulation of this element in soil–plant systems are key factors that drive its transfer along the food chain (Suhani et al. 2021). Comparing soil metal concentrations with other studies (Table 3), in some cases our study reported high levels of Hg, Cd, As and Fe. Accumulation for Cd in plants was highest in their order in *C. peltata* > *P. fasciculatum* > *T. rosea* > , As *P. fasciculatum* > *T. rosea* and for Pb *P. fasciculatum* > *C. peltata* > *T. rosea*. Research has indicated that the accumulation and translocation of Cd in particular may be determined by the degree of contamination and the type of plant (Bonanno et al. 2018; Pietrzykowski et al. 2018). Additionally, Pb and Cd exhibit higher accumulation in plant roots, with minimal content found in aerial parts (Sassykova et al. 2020).

**Table 3:** Comparison of potentially toxic metal concentrations (in mg/kg) in contaminated soils in different parts of the world

Country	Cd	Fe	Pb	Cu	Zn	Mn	Hg	As	Reference
Egypt	4.04	19.9	0.77	259.6	579.7	1.46			Eid and Shaltout (2016)
Saudi Arabia	0.8	2275	450	20	68	531			Eid et al. (2019)
Brazil							0.065		de Freitas et al. (2024)
Brazil			1.700						Paes et al. (2023)
India	3.3		126.6	52.3	84.9	303.9		354.1	Verma et al. (2021)
India	7.63	18.157	104.5	348.08	286.3	444.9		11.61	Kumar et al. (2019)
Iran	4.28	24.34	351.2		453.9				Hosseinniaee et al. (2022)
Iran		32466		488.4	195	459.5			Nouri et al. (2009)
China							0.305		Li et al. (2017)
China	0.24		28.86	28.60	77.94		0.08	13.1	Liu et al. (2020)
Poland	0.9		72.4		217.2				Pietrzykowski et al. (2018)
Poland	0.29		17.78	8.27	55.07				Korzeniowska and Dorocki, (2024)
Perú	1.143		2.523	273.8	287.2			287	Cruzado-Tafur et al. (2021)
Perú							7.26		Quispe et al. (2022)
México			1.40				279.4	14.1	Saldaña-Villanueva et al. (2022)
México	1.2		16.0	2.0	81	289		36.0	Franco-Hernández et al. (2010)
Colombia	0.52		2.03				0.06		Durante-Yánez et al. (2022)
Colombia							6.32		Marrugo-Negrete et al. (2016)
Colombia		21.1		8.10	805.3	96.6			Martínez and Marrugo-Negrete (2021)
Colombia	0.035		0.066	1004	1218		0.117		Marrugo-Negrete et al. (2017)
Colombia	0.21		9.42	11.28	31.93	110.5			Trujillo-González et al. (2023)
Colombia	1.7	34.5	0.2	0.03	0.1	0.8	18.4	28.8	This study

Total Hg concentration was higher in the leaves (3.5 mg/kg) than in the roots (2.8 mg/kg) (Table 2). Several studies have indicated the opposite, with Hg levels higher in leaves than in roots. When plants are contaminated through the soil, concentrations are generally higher in the roots than those found in the soil. Unlike when the Hg content is higher in the aerial parts, especially in leaf tissues, it is assumed that air could be the main source of contamination (Browne and Fang 1978; Li et al. 2017; Lindberg et al. 1979; Mikołajczak et al. 2017; Niu et al. 2013). However, some plant structures play a critical role in the Hg cycle, such as the needles of conifers (e.g. *Picea abies* (L.) H. Karst., and *Pinus sylvestris* L.) (Bishop et al. 1998). This evidence reinforces the hypothesis that species and metal type can largely explain variations in the ability of plants to accumulate metals (Barthwal et al. 2008; Fernández-Martínez et al. 2015). However, the positive correlation observed between Hg concentrations and plant roots (Table S1) to some extent reveals the incidence of atmospheric absorption on the accumulation and distribution of this element in plants. In the case of Hg, plant leaves can accumulate atmospheric Hg, especially elemental gaseous Hg (Hg(0)), which occurs as a result of several processes, including net uptake and fixation. The combination of these processes greatly affects foliar Hg bioavailability, translocation and the distribution of this element throughout the plant, in particular parts areas. Atmospheric Hg is retained within the foliage after stomatal or non-stomatal uptake. Subsequently, foliar Hg volatilizes after reduction, is expelled by fall, and is then deposited on the soil where it intervenes in the leaf litter and can be retained within the vegetation for long periods of time (Liu et al. 2022). In addition, tests of the linear relationship of Hg concentrations between air and leaves indicate that Hg accumulation in leaves is strongly influenced by atmospheric Hg, while in roots it is more affected by Hg transport in soils (Niu et al. 2013).

Similarly, atmospheric deposition of Pb is also crucial for understanding its accumulation in plants. This metal is mainly fixed on leaves through aerosols or fine dust particles suspended in the air, which are then mobilized by air masses (Fan et al. 2021). Free Pb ions are absorbed directly by plants, either by capillary action or by cellular respiration from the air (Collin et al. 2022). Lead sulfide (PbS) particles deposited on the leaf can be retained in leaf folds. When trapped in the folds, these particles can undergo important biochemical transformations due to their high reactivity and form new compounds with high Pb contents (Batonneau et al. 2000; Choël et al. 2006; Ur Rahman et al. 2024).

This process explains to some extent the high concentrations in the aerial parts of plants, although this may vary according to the type of plant. In addition, foliar deposition of Pb may be higher than the amount absorbed from the soil (Collin et al. 2022; Uzu et al. 2010).

We also examined the metal accumulation and translocation capacity of the collected plants. The overall value for BCF for Hg was 2.6 and 1.9 for TF, As (BCF 10.2, TF 2.1), Fe (BCF 18.2, TF 5.8) and Mn (BCF 2.9, TF 3.1). Several plants including *C. annuum*, *G. sepium*, *C. peltata and T. rosea* showed values exceeding one for both factors, suggesting their potential inclusion in the hyperaccumulator category for effective soil rehabilitation. The obtained results align with findings from other studies conducted in the surrounding landscapes, indicating that *C. annuum*, *S. alata*, *C. peltata* are identified as promising candidates for soil restoration in Colombia (Marrugo-Negrete et al. 2016; Vidal et al. 2010).

Criteria for evaluating phytoremediation potential indicate that those plants with BCF and TF values above one could be key to driving phytoremediation at contaminated sites (Hosseinniaee et al. 2022; Sun et al. 2016). A TF above one may also be a particular characteristic of metal-accumulating and excluding plants when their value is below one (Singh et al. 2010). For example, the low BCF values recorded in *A. muricata* for Hg and *C. peltata* for Cd (Fig. 3) suggest that these plants may be considered Hg and Cd excluders, respectively (Kafle et al. 2022; Sun et al. 2016). Interestingly, when contrasting the BCF and TF values obtained in our study, considerable variability was observed among the plant species studied. Some findings were consistent with our study, while others presented discrepancies. For example, for *C. annuum*, BCF and TF values were < 1 for Pb, which is consistent with the reports of Sun et al. (2016) for the same metal in *Solanum nigrum* L. (BCF: 0.25, TF: 0.09), both species belonging to the same botanical family. In contrast, *P. fasciculatum* was another species with a high TF value, reaching 3.1 for Pb and 1.5 for Cd. These values exceeded the maximum TF values for Pb (0.27) and Cd (0.12) reported by Salas-Moreno and Marrugo-Negrete, (2020) in an experimental unit with mining soils.

In the same line, BCF (0.83) and TF (1.19) values for Hg reported by Marrugo-Negrete et al. (2016) for *C. annuum* in the same study region are lower than those found in our research, which demonstrates a higher relative capacity of *C. annuum* to accumulate (1.93) and translocate (1.44) Hg. The same authors report BCF (0.73) and TF (0.43) values for *T. rosea*, lower than those reported in the present study (BCF: 2.38, TF: 1.99). These similarities and differences suggest that species may be employing differential mechanisms of adaptation to environmental conditions at each site. Furthermore, such variability may be related to the bioavailability of elements, state of disturbance of the ecosystem, edaphic factors (e.g. pH, organic matter), and the interaction between elements (Bishop et al. 1998; Gajić et al. 2018; Kafle et al. 2022; Krämer 2010).

The PCA1 reflected the highest load for Hg, with all plants, except *M. paradisiaca*, *P. fasciculatum*, *Bixa orellana* L., *P. guajava*, *T. rosea*, *M. citrifolia* and *C. peltata* showing a positive inclination towards this metal. These results are mainly attributed to the widespread dispersion of Hg in the environment, along with the responsive mechanisms of plants (Meyer et al. 2023). On the other hand, CA revealed that Cu and Zn had similar association with each other and probably derived from a common source, while Cd, Pb, Mn and Cd formed a distinct group (Sahoo et al. 2018).

It is true that, although all the plants investigated showed the capacity to grow in contaminated soils, there were notable differences in their capacity to remediate or clean those soils of contaminants with metals or other toxic compounds. These differences in phytoremediation potential suggest the intervention and functionality of the bacterial communities present in the soil (Paes et al. 2023). The efficiency of metal uptake and extraction by plants is regulated to some extent by enzymatic activities occurring in the soil, which are carried out by different beneficial microorganisms associated with the rhizosphere. Some species of the genera Pontibacter, Lysobacter and Kaistobacter can enhance plant growth and the uptake of specific metal ions and cations. In addition, they can secrete enzymes that help solubilize minerals present in the soil, including metals, making them available to plant roots (Lin et al. 2021).

For example, in the plant species *Arundo donax* L. it has been demonstrated that some bacteria such as *Agrobacterium sp* and *Stenotrophomonas maltophilia* Palleroni & Bradbury can substantially increase its growth and phytoaccumulation capacity of As. In addition, at concentrations of 2.0, 10.0 and 20.0 mg/L NaAsO_2_ the plant shows no signs of toxicity, on the contrary, it shows a slight increase in the biomass of stems and leaves (Guarino et al. 2020).

Another plausible explanation for a plant's remediation capacity is the abundance of genes related to the synthesis of specific proteins, such as P-type ATPase C, which is responsible for transporting metals such as Mg and Zn across plant cell membranes. In addition, it is suspected that metabolic pathways related to plant signaling compounds and energy-rich nucleotides may enhance the stress protection and growth capacity of plants in altered environments (Xiao et al. 2022).

In the case of *P. fasciculatum* a species recorded in our study is a plausible example of Pb phytoextraction. This plant demonstrates tolerance and capacity to extract this metal in soils heavily impacted by mining. The plant also contributes to improve soil pH and organic matter, two crucial factors in the uptake and accumulation process. Both factors (i.e. pH and organic matter) under the right conditions can favor metal uptake and accumulation. In addition, it has been shown that the plant maintains its photosynthetic capacity, due in part to the plant's enhanced antioxidant defenses, which help protect and repair proteins and transduction signals to coordinate physiological responses to metal-induced stress (Salas-Moreno et al. 2022).

Therefore, the phytoremediation potential of *P. fasciculatum* has a positive and promising effect for the restoration of soil contaminated with Pb, a highly toxic metal, and according to research, this metal can remain in the soil for at least 5000 years, and remain in high concentrations for a long time, even after the application of remedial measures including the application of sludge (Jabeen et al. 2009; Purves 1986).

On the other hand, the combination and interaction between hyperaccumulator plants and microorganisms is ideal to formulate effective remediation strategies for mining-impacted areas (Xuan et al. 2022). This is partly explained in that these plants tend to accumulate higher concentrations of metals in root and shoot tissues through general mechanisms such as metal cation uptake and the formation of metal-phytochelatin complexes (M-PC) or metal–ligand complexes that occur within cells (Lajayer et al. 2019).

In summary, behind the differences in the ability of plants to accumulate and distribute metals recorded in our study is the influence of several factors, including plant type, soil pH, cation exchange capacity, soil organic matter content and type of metal (Kafle et al. 2022; Nouri et al. 2009). As mentioned in a previous excerpt, Hg concentrations were higher in the leaves compared to the stem, for example. This in part may be related to the main function of stems to transport water and nutrients from roots to leaves via xylem and phloem tissues, which may explain the low Hg content in this organ. Consequently, fluid dynamics in the stem may probably be preventing Hg from accumulating in large amounts, favoring its transport to the aerial parts of the plant, such as the leaves (De Boer and Volkov 2003; Marrugo-Negrete et al. 2016).

In altered environments, plants can employ as an adaptive and response strategy to the toxic effects of metals and other harmful elements. One of these strategies involves the activation of mechanisms at the cellular level, such as the modification of plasma membranes and the expression of transporter proteins, such as metallothioneins (MTs) located in the vacuolar membrane. Many MTs have the function of exporting toxic elements to the vacuoles and sending them to the upper parts of the plant, allowing adaptation and survival in critical conditions (Gill et al. 2021). Additionally, it is true that acid pH favors the accumulation of metals including Hg in plant roots. This relationship is due to the fact that Hg tends to form more soluble chemical species in acidic media and to be more available for uptake by plant roots (Kafle et al. 2022; Krämer 2010; Sharma et al. 2023).

## Conclusions

The findings in our study offer valuable insights to enhance the list of potential plant species for the remediation of soils contaminated with toxic metals. This is particularly significant, as the identification of species capable of stabilizing and accumulating metals is a key challenge in phytoremediation for environmentally impacted landscapes. The median cumulative soil concentrations for As, Hg, Cd, and Pb were 28.8 mg/kg, 18.4 mg/kg, 1.7 mg/kg and 0.2 mg/kg, respectively. The overall Hg contents in plants were observed in a higher proportion in the leaves (3.5 mg/kg), while Pb and As contents were higher in roots with 17.7 mg/kg and 3.8 mg/kg, respectively. Median Pb in *P. fasciculatum* concentrations were 10.1 mg/kg in roots and 4.0 mg/kg in stems, and for Hg 0.3 mg/kg in roots and 0.2 mg/kg in leaves. For the same plant, As concentrations were 9.6 mg/kg in roots and 0.6 mg/kg in leaves. These results indicated that this particular species has a good capacity to extract toxic metals, especially Cd and As. Overall, the findings of this study suggest that *P. fasciculatum*, *A. muricata*, *M. citrifolia*, *G. sepium* and *T. rosea* are potential candidates for developing strategies aimed at rehabilitation soils contaminated with toxic metal(loid). This information provides a foundation for mining companies to integrate phytoremediation programs using native plants into their social and environmental responsibility initiatives. However, a more comprehensive understanding of soil-air-plant interactions is essential for these programs to succeed. Such insights will not only broaden the selection of suitable plant species but also enable the integration of complementary remediation techniques, resulting in faster, more cost-effective, and sustainable outcomes.

## Supplementary Information

Below is the link to the electronic supplementary material.

Supplementary file1 (DOCX 13.8 KB)

## Data Availability

All authors declare that all data and materials as well as software application or custom code support their published claims and comply with field standards.
