# Supplementary material for: Exploring the phytoremediation potential of plant species in soils impacted by gold mining in Northern Colombia
Source: Environ Sci Pollut Res Int. 2025 Jan 21;32(7):3795–808. doi: 10.1007/s11356-024-35853-8 (PMC11835935; doi:10.1007/s11356-024-35853-8)
Supplement: Supplementary file 1 — Supplementary file1 (DOCX 13.8 KB) [file 11356_2024_35853_MOESM1_ESM.docx]

**Table S1.** Pearson's correlation coefficient between the natural logarithm of metal concentrations in soil (mg/kg) and roots considering all plants.

| Metals | Pearson r | *p* value |
| --- | --- | --- |
| Hg | 0.431 | 0.0122 |
| Cd | 0.681 | 0.0147 |
| Cu | 0.768 | 0.0057 |
| Zn | 0.760 | 0.0041 |
| Mn | 0.719 | 0.0084 |

*p*: significance of correlation.
